# Graphical user interface for simultaneous profiling of activity patterns in multiple neuronal subclasses

**DOI:** 10.1016/j.dib.2018.07.069

**Published:** 2018-08-07

**Authors:** R. Ryley Parrish, John Grady, Neela K. Codadu, Claudia Racca, Andrew J. Trevelyan

**Affiliations:** a Institute of Neuroscience, Medical School, Framlington Place, Newcastle upon Tyne NE2 4HH, UK; b Kinghorn Centre for Clinical Genomics, Garvan Institute, 384 Victoria Street, Darlinghurst, Sydney, NSW 2010, Australia

**Keywords:** Pyramidal neurons, Interneurons, Glia, Astrocytes, MATLAB, Immunohistochemistry, Ca^2+^ imaging

## Abstract

We provide notes on how to use a graphical user interface (GUI), implemented with MATLAB, for aligning imaging datasets of biological tissue. The original use was for matching two imaging data sets, where one set was taken of the living preparation and another was taken post-fixation and following immunohistochemical processing. This technique is described in detail in an accompanying paper (Parrish et al., [1], where we also include information about the experimental procedures, and examples of the usage of the GUI.

**Specifications Table**

**Table:** 

Subject area	*Neuroscience*
More specific subject area	*Analysis of the cell class involvement in neuronal network behavior, assessed using Ca*^*2+*^*network imaging*
Type of data	*Images, software*
How data was acquired	*Microscopy and electrophysiology*
Data format	*analysed*
Experimental factors	*None*
Experimental features	*A description of how to use a custom-written image registration software, for matching functional imaging data with other images of the same tissue stained for specific cell classes.*
Data source location	*Newcastle upon Tyne, UK*
Data accessibility	*We provide software, implemented using MATLAB*
Related research article	*Parrish RR, Grady J, Codadu NK, Racca C, Trevelyan AJ. Simultaneous profiling of activity patterns in multiple neuronal subclasses. Journal of Neuroscience Methods, 2018; In review*

**Value of the data**

• Ca^2+^ imaging is a widely used experimental techniques for investigating neuronal firing in small to medium size networks of neurons.
• A major limitation of this technique is the inability to identify subclasses of neurons.
• We solved this problem by matching functional imaging of live tissue on to images of the same, fixed tissue, stained for different immunohistochemical markers.
• We provide the software for matching the live and fixed tissue images, and describe how to use it.

## 1 Data

We provide notes on how to use a graphical user interface (GUI), implemented with MATLAB, for aligning imaging datasets of biological tissue. The original use was for matching two imaging data sets, where one set was taken of the living preparation and another was taken post-fixation and following immunohistochemical processing. This technique is described in detail in an accompanying paper [1], where we also include information about the experimental procedures, and examples of the usage of the GUI.

## 2 Experimental design, materials, and methods

We describe how to use a graphical user interface (GUI), implemented on MATLAB, which was developed for aligning live and fixed imaging data based on sets of cellular landmarks that can be identified in both data sets. The original motivation for doing this was to identify different neuronal classes having performed Ca^2+^ network imaging of activity of up to several hundred cells simultaneously, while the brain slice was experiencing epileptiform activity [2], [3]. The details of these experiments are described in an accompanying paper [1], but it is helpful to describe this briefly here too. In short, we acquired Ca^2+^ network imaging data from a single plane of a living brain slice. One or more neurons in the field of view were filled with Alexa Fluor 594 and biocytin, to allow immediate visualization of the dendritic tree in the live imaging (Alexa Fluor 594) and the potential for subsequent visualisation once fixed (biocytin). The astrocyte population was also stained using the glia-specific vital dye, Sulforhodamine 101 (SR101) [4]. Z-stacks were taken of the living tissue of the Ca^2+^ dye labelling, the filled cells and also the glial labelling. The brain slice was then fixed and stained for different immunohistochemical markers. The specific purpose of the software we describe here is then to realign the live imaging data to the fixed imaging, so that we can ascribe immunohistochemical markers, from the fixed imaging data sets, to the cells seen in the live imaging.

The realignment process is required because the fixation process generally introduces a small amount of shrinkage and distortion of the tissue (on average 11% (*n* = 5 slices); range between 5% and 30%). The realignment process creates a distorted (“transformed”) new version of only one of the datasets – this is the “source” image, which is matched to the non-distorted “target” image. For our data, the live imaging was designated as the target images, and the fixed images were the “source images”; the reason for this is that the critical data set is the living Ca^2+^ imaging time series, which provides the functional context of the subsequent interpretation of the immunohistochemistry labellings. Thus, it is most helpful to keep the original dimensions of the live imaging data sets, and to reshape the fixed images to match on to the live imaging.

The software is run as a Graphical User Interface (GUI) on the MATLAB platform (Mathworks). The accompanying matchgui.m file has been tested in release versions through to R2016b. The matchgui.m file should be located in a folder that is identified as a set path, using the MATLAB addpath command (e.g. *addpath(genpath(׳D:/MatlabScripts׳))*). The image files (we always used either single image tifs, or tif stacks, but other formats could also be loaded) should be located in the “Current Folder”. This is also important when subsequently retrieving old matches, because the saved alignments reference the original tif stacks, and the program will not work if it cannot find these. In the description below, the specific Matlab commands are written italicized.

## 3 Using the GUI

### 3.1 Loading in imaging data

**Fig. 1:**
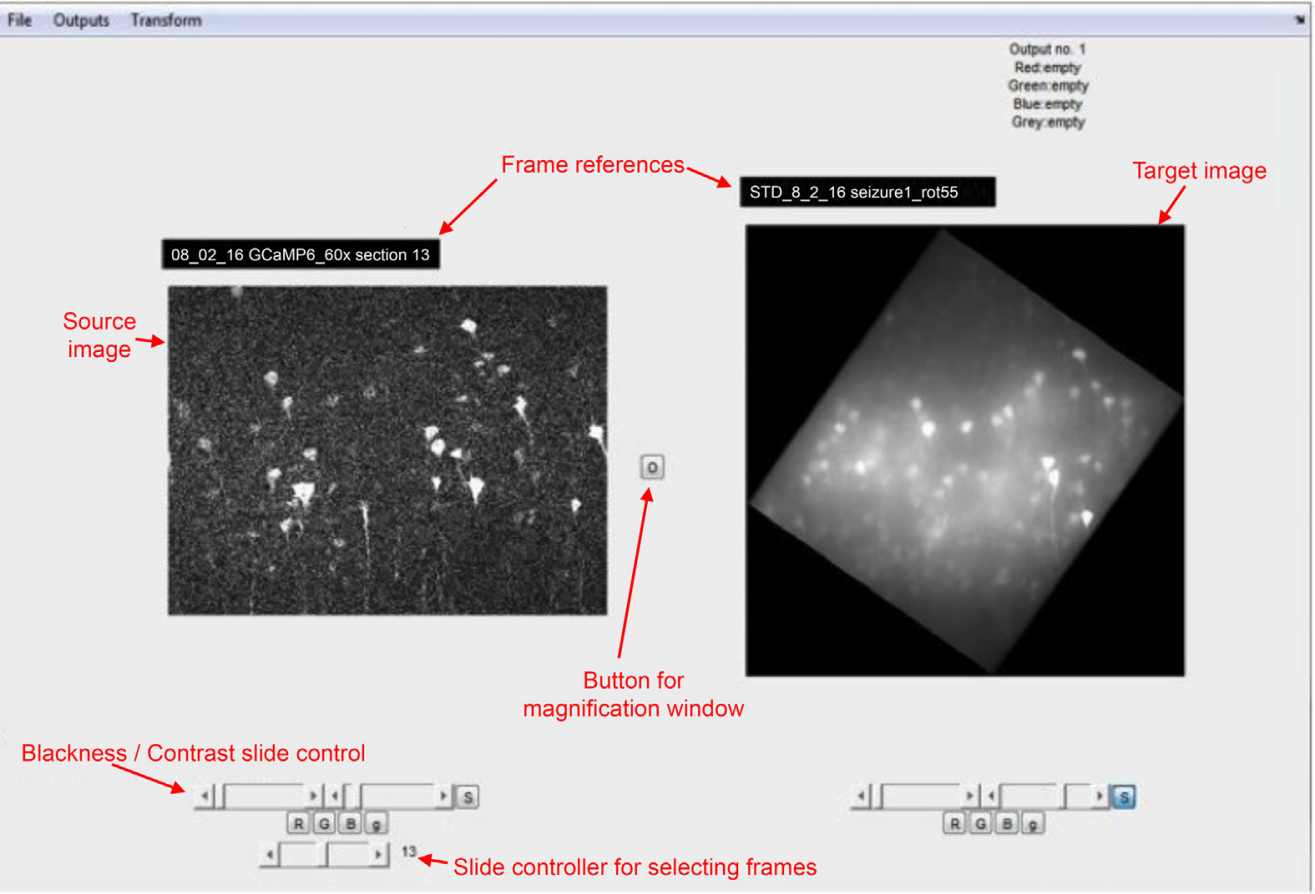
Annotated screenshot of the Graphical user interface. View of the initial, main screen for the GUI.

The GUI is started by typing *matchgui* in the MATLAB command window (Fig. 1).

### 3.2 Loading images into the GUI

The imaging data are loaded using commands in the File menu at the top left of the GUI. Under the File tab, the “Add Source Image(s)” should be used to load the fixed image stack. Note that many different fixed stacks can be loaded into the source image files. Following that, use the “Add Target Image(s)” to upload the live imaging as a projection of frames or a single frame. It is of course imperative that all the fixed images are aligned with each other, and likewise, that all the live tissue images are co-aligned, otherwise the matching process based on one set of images will not translate to a different set.

When loading up stacks of source images (the images that will be reshaped), the GUI provides two different options, either to load up the individual frames of the stack, or to load it up as a block. The first option (answer “Yes”, which loads up individual frames of the stack) is essential for the process of identifying corresponding landmarks in the source and target imaging datasets. The second option (answer “No”), loading as a stack, allows this entire stack to be subsequently selected as a single entity for a transformation, but note that this option cannot be used for the matching process, because the individual tifs within the stack cannot be accessed. Thus, one often needs to upload stacks of source images twice, using both options. In short, the “individual frames” option is for making the landmark matches (step 2), whereas the “block” option can be used for generating outputs (transformed images; step 3). Note, however, that all the images within either the target or source groups must have the same *xy* dimensions, although the target and the source stacks typically have different dimensions.

For our experiments, we loaded up images as follows:

1. Source files (fixed imaging)
  a. Stack of biocytin imaging – specify as individual images (answer “Yes” at menu)
  b. Stack of biocytin imaging – specify as a stack (answer “No” at menu): this is for generating the output!
  c. Stack of NeuroTrace - individual images
  d. Stack of NeuroTrace - single stack (for output purposes) – note that if you already know that the activity data are incorporated within a narrow range of the stack, one can create a stack subrange for this purpose (i.e. you don’t need to use the whole stack)
  e. Stack of glia (can be useful if blood vessels provide landmarks)
  f. Stack of any other immunohistochemical labelling – as single stack for outputting

2. Target files (live imaging)
  a. Stack of biocytin imaging
  b. Compressed stack of biocytin (for output)
  c. Ca^2+^ labelling stack (can also be useful for blood vessels)
  d. SR101 stack
  e. Single image of compression of activity patterns – this will be equivalent to a single plane of view in the Ca^2+^ imaging stack.

Note that on occasions the live imaging sets may be slightly shifted relative to each other (if for instance, one moves the stage and does not quite correct it, or if the slice shifts in the bath). These must be put back into register before loading.

### 3.3 Identification of paired, matched landmarks

**Fig. 2:**
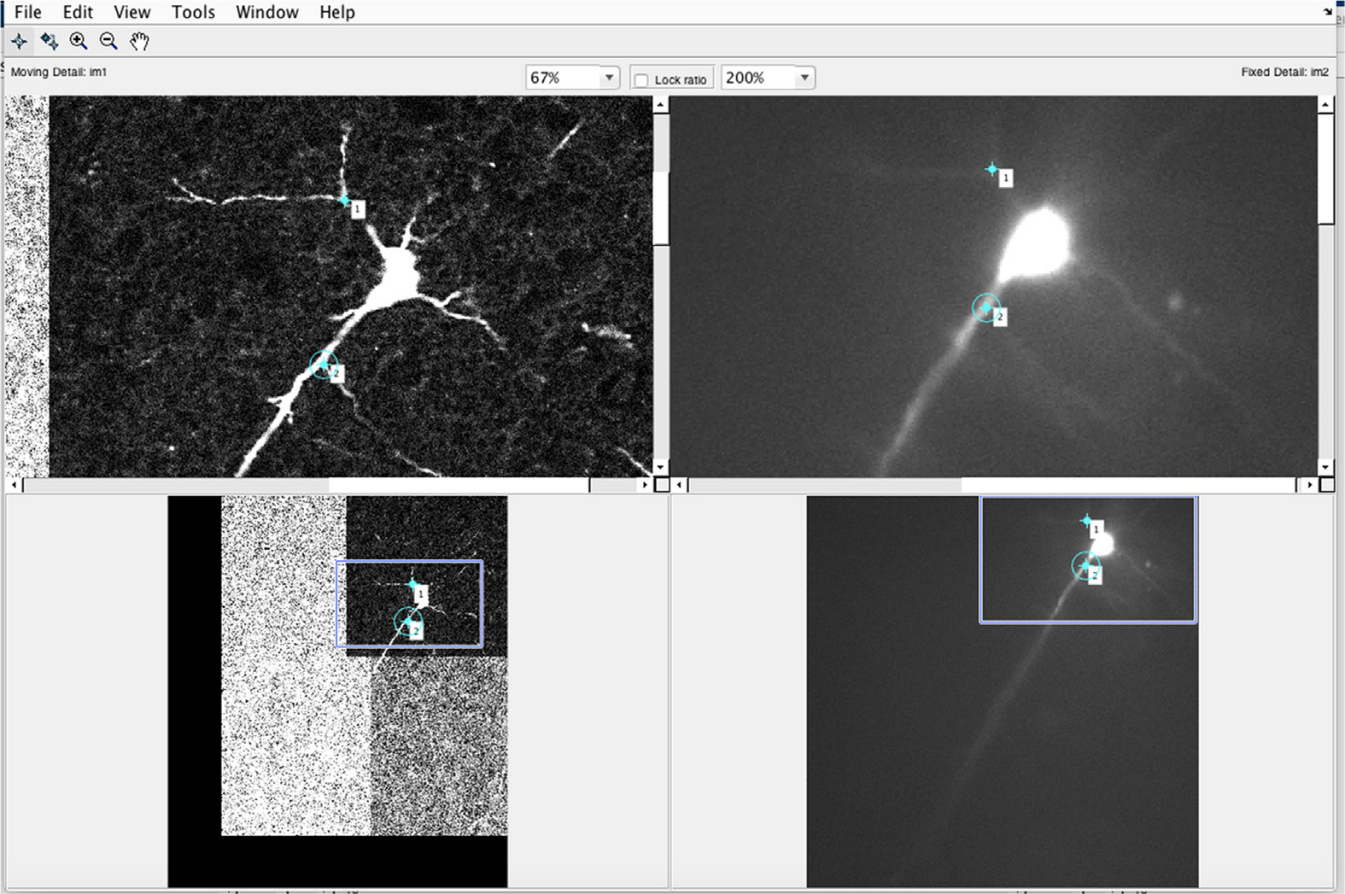
Screenshot of the magnification window. The left images are of the fixed biocytin-processed labelling of the filled cell, and the right images were of the living cell, filled with Alexa Fluor 594. One can see the same dendritic branch points on both images, and two have been marked as matching landmarks. Note, however, that because the live image has the contrast gain shifted to allow one to see the fine dendritic structure, this saturates the soma and appears to make it look larger, and potentially obscuring potential branch points. The contrast can be adjusted in this window to allow these other branches to be identified. The magnification of the two images can be set independently, and the exact location of the magnified field of view can be shifted by dragging the box on the lower images. Both these features help with identification of landmarks, guided by clustering patterns of the landmarks. These show the first two landmarks identified, but the window will also show landmarks from other frames in the stacks.

**Fig. 3:**
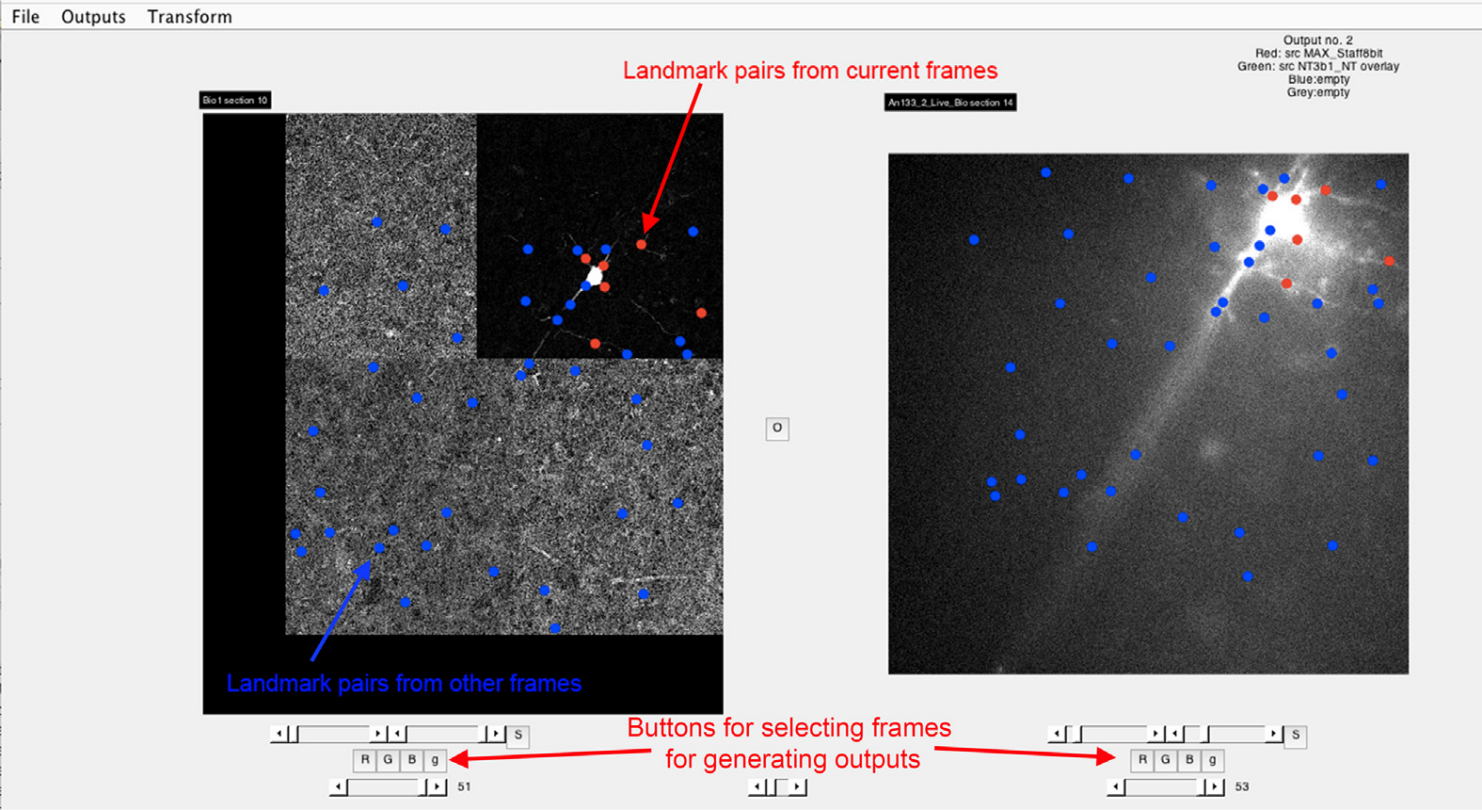
Screenshot of the main GUI screen showing landmarks. When the magnification window is shut, the landmarks are displayed on the full image in either red or blue. Red designates that the landmarks were made from that frame of the stack, whereas blue indicates that the matching was made from a different frame. If one clicks on a specific dot, the GUI jumps to the relevant matched frames, allowing that pair to be edited, if necessary. Note, also that the left “Target” image is a montage. Montages can be used perfectly well, as long as the *xy* dimensions are constant for all the images in that grouping.

Next, one identifies corresponding landmarks in the source and target imaging sets. The easiest landmarks to identify are the dendritic branch-points of any filled cells (Fig. 2, Fig. 3). These are then marked in both the target and source, using a second window, which is opened by first clicking the button between the two images (Fig. 1, labelled “Button for magnification window”), and which allows differential magnification and moving of the current images. Move the cursor to the landmark in one image, left click, and then move to the same landmark in the second image and left click again. One can easily edit these landmark flags, either by moving the cursor to a particular marker, and holding down the left cursor, which allows that marker to be moved, or alternatively, using the Edit menu (top left), which allows one or both of the active marker pair to be deleted. Once the landmarks have been identified within the current window that can be closed in order to examine other frames in the stacks. Note that one cannot scroll to other frames while this magnification window is open. The matched landmarks will now appear in the control point transformation window, initially seen as red markers, but these then turn blue as one moves onto a different image in the stack – thus the red/blue colouration denotes whether one is at the same plane that provided the original match.

As one builds up an array of matched paired points, the constellation of these points provides some guidance about the accuracy of the matches. This is more relevant when one is pairing cell bodies, such as with the glial labelling, which do not provide such unique features as the dendritic branches. The best transformations are achieved when the constellation of landmarks is distributed throughout the entire field of view. Realistically, this never quite happens, and a more usual state of affairs is that one has good coverage of only a portion of the field of view. Since the most accurate landmarks are from dendritic branching points, it stands to reason that filling two or three neurons in the field of view is likely to give the best transformations. Glial matches on the other hand can be more difficult, mainly because these provide less structural information than there is for the extensive dendritic arborisation.

As the number of matched landmark pairs increases, the clustering patterns in the source and target images can themselves become a guide for identifying further landmark pairs, or for identifying errors. Correcting these errors is only possible from the frame in which they were originally marked, but clicking on one of the landmark dots takes you immediately to that frame.

### 3.4 Generating transformed images

Once one has accumulated a set of landmark pairs, which optimally gives an even coverage of the field of view of the target image, then one can transform the source onto that target. To do this, select the relevant transformation by clicking on the “Transform” menu option (top left), and then click on Output, and select “new”. Next, scroll through the target images to find those that need to be transformed. These are selected by clicking on the R, B, G, g (“Red”, “Blue”, “Green” and “grey”), which designates the colour for that particular image in the transformed output. Once selected, the reference for the output image is shown at the top right. One can select up to 4 images in an individual transform, but the transformation can still be done if fewer are selected. The transformed image is generated by selecting “Generate current output” from the Outputs menu. Entire stacks of images can be transformed at once, if the stack option was selected when loading images. One can, however, load up a new stack even after the landmark matching. Note also that images selected from the source stack remain exactly the same, which may seem to be not very useful, but the utility of this comes in helping identify the correct z-plane.

### 3.5 Identifying the correct z-plane

In our experiments, there was typically a single particular depth of imaging that was more important than any other, namely, the focal plane at which the functional Ca^2+^ imaging data were collected. The original motivation for designing this software was to enable us to identify subclasses of cells from this data set of functional firing patterns, and thereby start to understand the roles that different neurons play in particular network events. It is helpful therefore to have an image of the Ca^2+^ labelling at this plane, within the source stack, and, ideally, one which shows large numbers of cells. Some dyes show little signal at rest, meaning that at these times, most cells are invisible and so a single live imaging snapshot may not suffice for this. However, one can generate a pseudo-representation of the plane by creating a standard deviation z-compression of the movie, which can highlight the cells that show most intense fluctuation. This works particularly well for epileptic activity, which can involve most if not all neurons within the network. Other Ca^2+^ dyes are visible at resting membrane potential (if the *K*_d_ is low enough), while others such as Fura-2, when illuminated above its isosbestic point, show an inverse signal, with high fluorescence at low Ca^2+^, which is then quenched by increases in Ca^2+^ secondary to neuronal firing. For these dyes, a single snapshot at the relevant imaging plane will suffice.

**Fig. 4:**
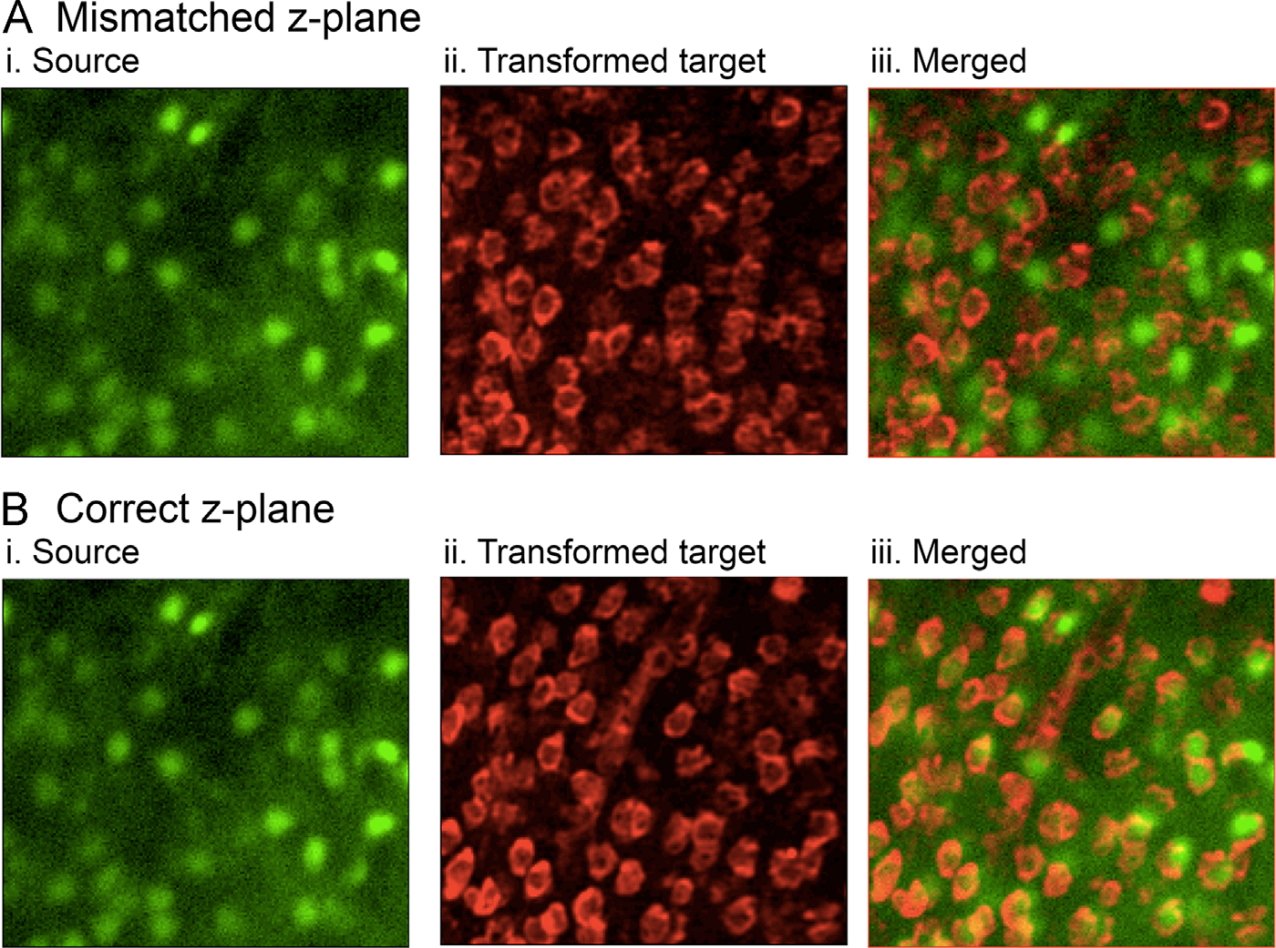
Identifying the correct focal plane. Two different “planes” of an output stack generated from a single “Source” image of a standard deviation z-compression from a time series of Ca^2+^ network imaging of epileptiform activity, and a transformed stack from the Target set, of the NeuroTrace stain. At the incorrect plane, the two images do not correspond, whereas at the correct plane, they do.

This “Source” image should then be selected for one colour in the output, and then select from the “Target” images a stack of the neuronal cell bodies. We have used the Neurotrace stain to this end. Generating an output from this pairing will create a tif stack, in which each successive, transformed image from the Target stack is paired with the same image, selected from the Source stack. As one scrolls through this, there is a complete lack of correspondence of the two images, except at the correct plane of focus, which can thereby be easily identified (Fig. 4). Having identified the correct plane, one can then start to subclassify the neuronal population at that plane from the fixed imaging stacks of the other immunohistochemical labellings.

### 3.6 Saving the matching data

At any stage, the matchgui process can be saved, using the File menu at the top left. This generates a mat file, which, when opened, contains all the requisite information in a structure called *g_data*. Note that the saved mat files from matchgui do not contain the actual images but rather, contain the file references to allow those images to be reloaded. This is important, because one must ensure that all the tifs are present in the current directory, when one reloads the file in matchgui (File menu, Load). One can then add further images, and generate new transformed outputs, accordingly.
